# Enhanced Energy Localization in Hyperthermia Treatment Based on Hybrid Electromagnetic and Ultrasonic System: Proof of Concept with Numerical Simulations

**DOI:** 10.1155/2017/5787484

**Published:** 2017-08-01

**Authors:** N. Nizam-Uddin, Ibrahim Elshafiey

**Affiliations:** Electrical Engineering Department, King Saud University, Riyadh, Saudi Arabia

## Abstract

This paper proposes a hybrid hyperthermia treatment system, utilizing two noninvasive modalities for treating brain tumors. The proposed system depends on focusing electromagnetic (EM) and ultrasound (US) energies. The EM hyperthermia subsystem enhances energy localization by incorporating a multichannel wideband setting and coherent-phased-array technique. A genetic algorithm based optimization tool is developed to enhance the specific absorption rate (SAR) distribution by reducing hotspots and maximizing energy deposition at tumor regions. The treatment performance is also enhanced by augmenting an ultrasonic subsystem to allow focused energy deposition into deep tumors. The therapeutic faculty of ultrasonic energy is assessed by examining the control of mechanical alignment of transducer array elements. A time reversal (TR) approach is then investigated to address challenges in energy focus in both subsystems. Simulation results of the synergetic effect of both modalities assuming a simplified model of human head phantom demonstrate the feasibility of the proposed hybrid technique as a noninvasive tool for thermal treatment of brain tumors.

## 1. Introduction

The efficacy of hyperthermia thermal treatment depends on localizing the energy to increase the temperature of the cancerous tissue into the range 42°C–45°C, while preserving healthy tissues at normal levels. This temperature elevation increases the effectiveness of conventional techniques such as radiotherapy and chemotherapy [1]. In order to achieve this therapeutic temperature, the patient is locally submitted to an electromagnetic (EM) or ultrasound (US) field. This therapeutic temperature is correlated to specific absorption rate (SAR), which quantifies the amount of heat accumulated in human tissue and is related to tissue characteristics and electric or pressure field intensity depending upon the number of heating sources and frequency of operation [2].

An effective thermal treatment however depends upon several factors including treatment of deep-seated tumors, energy localization at tumor site, high resolution imaging system to monitor the therapeutic process, and the noninvasive nature of the treatment. Energy localization at the tumor site without excessively heating the surrounding healthy tissue in the head region by means of external EM and ultrasound sources is the primary subject of this paper.

EM techniques are widely used in thermal treatment systems [3–5]. EM use in hyperthermia treatment however suffers from some discrepancies related to energy penetration and focusing capability in targeting deep-seated tumors. Novel techniques have been reported to overcome these limitations such as the introduction of nanomaterials for improving EM therapy treatment [6], the use of fullerene in enhancing microwave therapy [7], and adopting magnetic nanoparticles to enhance treatment performance [8]. Some of these findings however find limitations related to the requirement of some noninvasive treatment procedures. This research utilizes the noninvasive treatment capabilities of ultrasound techniques to improve the efficacy of EM hyperthermia by exploiting the synergetic effect of both modalities.

Various techniques have been reported in the literature to localize EM energy utilizing phased arrays [9], beamforming approach [10], and multifrequency techniques [11]. In addition, optimization techniques have been reported using eigenvalue analysis [12], genetic algorithms [13, 14], and temperature control feedback approaches [15]. Focusing of energy however becomes more challenging when treating cancerous tumors in the head region due to the tissue-bone interface in the human skull. Since the penetration of EM energy is more pronounced in skull bone as compared to ultrasound, therefore, various techniques can be opted for to achieve energy localization. These include three-dimensional time reversal technique in [16], waveform transmitting diversity approach for wideband MIMO radar [17, 18], and nonuniform and short inverse FFT based wideband beamforming method [19]. Compressed sensing beamforming presented in [20] and sparse array based wideband beamforming discussed in [21] can be implemented to improve energy localization. In this work, we propose a multichannel wideband EM hyperthermia system to focus energy and devise a genetic algorithm (GA) as a heuristic optimization technique to enhance SAR accumulation.

Focusing of acoustic energy can be accomplished using reflectors [22], acoustic lenses [23], spherically curved transducers [24], or phased-array applicators [25]. It also includes an amplitude modulation technique for ablation in soft tissues [26] and multifrequency focused ultrasound [27–29] approaches. As acoustic waves propagate towards the head region, they suffer from absorption and reflections at the tissue-bone interface, thus making energy localization more challenging. The high impedance of the skull bone induces strong reflection of the wavefront. Moreover, difference in skull thickness and speed of sound heterogeneities inside the skull causes the focal point of the energy envelope to shift and thereby destroy the energy focus [30]. Among several challenges in clinical US therapy is the noninvasive transcranial delivery of US energy inside the human skull and enhancing energy localization in the tumor region without damaging the surrounding healthy brain tissue. Various studies have been performed to investigate the feasibility of focusing ultrasound energy inside the human skull. These include a noninvasive approach in [31],* ex vivo* heterogeneous medium optimization in [32], amplitude and phase correction in [33], phased array in [34], cylindrical array applicator in [35], and time reversal methods in [36–38]. Other advanced techniques depend on* in vivo* phase aberration correction techniques in [37, 39], along with image guided therapeutic approaches [40–42].

In this work, the focusing of ultrasonic energy is examined using phased array, utilizing the mechanical alignment of the transducer array elements with respect to the known tumor location. A time reversal (TR) based focusing approach is also investigated for acoustic as well as EM energy. The subsequent sections explain the proposed hybrid system, problem formulation of the methods, and techniques used to increase the efficacy of both treatment modalities. Simulation results are then presented, followed by illustration of thermal capabilities achieved by the synergetic effect of both modalities.

## 2. The Proposed Hybrid Hyperthermia Treatment System

In this communication, we propose a hybrid treatment system for treating cancerous head tumors. We propose a novel EM thermal treatment approach based on a multichannel and wideband system to enhance energy localization. The system is augmented by a US treatment subsystem to enhance energy localization. The two subsystems are described next.

### 2.1. Electromagnetic (EM) Therapy Module

**Figure 1:**
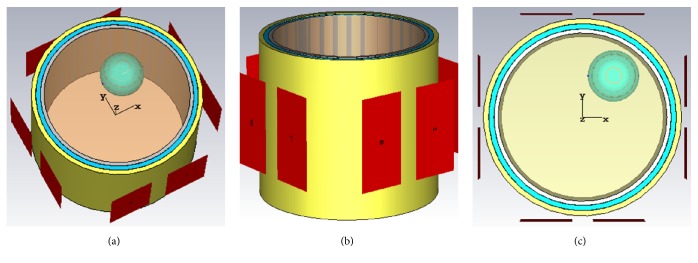
(a) Perspective view of the proposed multichannel wideband hyperthermia treatment incorporated with different head tissue layers; (b) side view indicating the arrangement of ports; (c) front view indicating the location of the tumor at top right.

To investigate the performance of electromagnetic (EM) thermal treatment, a multichannel wideband head phantom model is built using CST microwave studio [43]. This model consists of a four-layered cylindrical phantom of radius 10 cm to represent a human head. The inner cylinders of radii 8, 8.4, 8.9, and 9.4 cm depict brain tissue, gray matter, cerebrospinal fluid (CSF), and skull, respectively, as shown in Figure 1(a).

**Figure 2:**
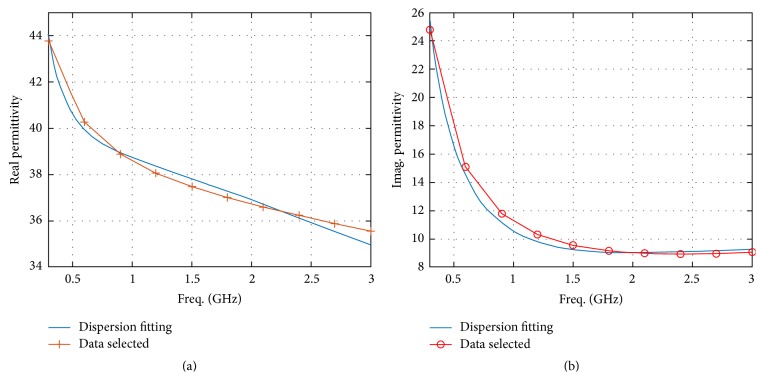
Frequency response of human brain tissue in terms of (a) real permittivity and (b) imaginary permittivity values.

The applicator ports surround the phantom as shown in Figure 1(b). The tumor is spherical in shape with a radius of 2.5 cm and is located at *x* = 3 cm, *y* = 4 cm, and *z* = 0 cm, as revealed in Figure 1(c), where the origin is set at the center of the phantom. The background material of the head phantom is set as water. The wideband dielectric properties of brain tissue are selected in accordance with [44–46]. Figures 2(a) and 2(b) represent the frequency response of brain tissue.

### 2.2. Ultrasound Therapy Module

**Figure 3:**
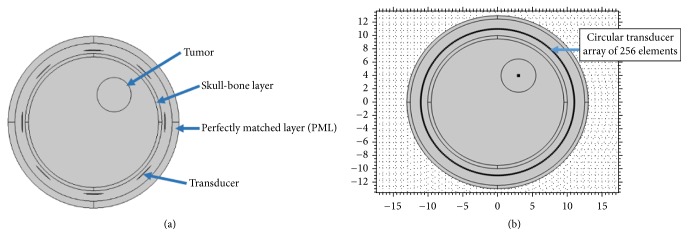
Ultrasound hyperthermia model of human head with embedded tumor at top right with (a) 8 transducers and (b) 256 transducers.

The ultrasound thermal treatment is analyzed by developing a 2D circular head phantom model in COMSOL environment [47]. A circular head phantom filled with brain tissue material is represented by a circle of radius 10 cm and is surrounded by an 8-element transducer array as shown in Figure 3(a). This initial model is upgraded by a circular array of 256-element transducers as illustrated in Figure 3(b) for TR focusing investigation. The tumor is circular in shape of 2.5 cm radius located at *x* = 3 cm, *y* = 4 cm, and *z* = 0 cm (same location as in EM hyperthermia). The bone is of 10.5 cm radius and has a thickness of 0.5 cm. Water coupling medium of 12.5 cm surrounds the skull bone. An extra layer of 0.5 cm around the water coupling medium is used for perfectly matched layer (PML) boundary conditions.

The acoustic properties of brain, skull bone, and water are frequency dependent and are derived from [48, 49]. We characterize our proposed ultrasound hyperthermia model for 0.5 MHz frequency and therefore name it reference frequency and study the results of insonation for other frequencies such as 0.05 and 0.1 MHz. The choice of low frequency values may not be ideal for clinically adopted US systems, but here we choose them to illustrate the effect of frequency on pressure maps. The acoustic properties for the reference frequency are given in Table 1.

Next, we present energy localization techniques for our proposed hybrid treatment model.

## 3. The Proposed Energy Localization Techniques

Owing to different dielectric and acoustic properties of our proposed research, it becomes challenging to focus energy and enhance energy localization. Therefore, to meet this challenge, we aim to implement the global optimization technique in the form of GA and a more robust technique such as TR. Initially, we present problem formulation for our proposed hybrid system by assuming circular array of *M* applicators/transducers located in a coupling or cooling medium surrounding the head region at locations **r**_*m*_  (*m* = 1,2,…, *M*). Let **x**_*m*_(*n*)  (*n* = 1,2,…, *N*) represent the EM/US signal transmitted by the *m*th applicator and *n* denote the discrete time sample of the excitation signal. The focused EM or US field at a location *r* inside the head can be described as

(1) $$\begin{array}{c} e\left(\;r,n\;\right)=\overset{M}{\underset{m=1}{\sum }}\Psi \left(\;f_{0},r_{m},r\;\right)x_{m}\left(\;n\;\right), \end{array}$$

where *f*_0_ is the operation frequency and Ψ(*f*_0_, **r**_*m*_, **r**) depicts the propagation mechanism in a heterogeneous medium.

The above equation can be written in matrix form as follows:

(2) $$\begin{array}{c} E=AX, \end{array}$$

where **A** is the *M* × *N* matrix substitutes for Ψ(*f*_0_, **r**_*m*_, **r**). **X** is the *N* × 1 matrix representing the complex excitation of the array elements. **E** is the *M* × 1 matrix that denotes the field intensity at a location *r*. An optimum value of **X** can be achieved by choosing appropriate antenna weights in terms of phase and magnitude and can be solved as

(3) $$\begin{array}{c} X=WA^{*t}{\left(\;AWA^{*t}\;\right)}^{-1}E, \end{array}$$

where **W** is *N* × *N* weighting matrix and **A**^**∗t**^ is the conjugate transpose of **A**. Designing an optimum **W** leads to localization of electromagnetic or acoustic energy.

The resulting optimized specific absorption rate (SAR) in EM at a location *r* can be written as

(4) $$\begin{array}{c} {SAR}_{opt}\left(\;r\;\right)=\frac{\sigma_{eff}\left(r\right){\left|E\left(r\right)\right|}^{2}}{2\rho \left(r\right)}. \end{array}$$

For US case, SAR can be quantified as

(5) $$\begin{array}{c} {SAR}_{opt}\left(\;r\;\right)=\frac{{\left|Q\left(r\right)\right|}^{2}}{\rho \left(r\right)}, \end{array}$$

where *Q*(*r*) = 2*αI*(*r*) is deposited acoustic energy at location *r*, *α* is the attenuation coefficient in the tissue, and *I*(*r*) is the ultrasound wave intensity.

The evaluation of the matrix **X** implies optimization of the proposed system. For EM hyperthermia, we propose genetic algorithm (GA), phase array processing, and time reversal techniques to achieve SAR optimization. For US, we emphasize phased-array and TR focusing techniques.

With the increase of the number of channels of the system and the number of subcarriers in each channel, the number of degrees of freedom becomes very high and the optimization process becomes computationally complex. Thus, a robust and computationally efficient algorithm is needed to address the complexity of such systems. Various techniques have been reported in the literature for SAR optimization in EM hyperthermia systems such time reversal [50], fixed-time topology [51], inverse multiphysics strategy [52], genetic algorithms in [13, 14, 53], eigenvalue based optimization in [12], projection algorithm in [54], and particle swarm optimization in [55]. We devise an optimization technique based on the genetic algorithm to reduce any possible hotspots. Additionally, time reversal (TR) focusing technique is adopted to minimize the computational complexity associated with GA implementation.

### 3.1. Genetic Algorithm Based SAR Optimization

GA techniques are used here to optimize the values of the magnitude and phase corresponding to each frequency subcarrier in all of the excitation channels. We propose wideband SAR optimization by defining the objective function given as

(6) $$\begin{array}{c} \xi \left(\;M,f,X\;\right)=\overset{M=8}{\underset{\forall f}{\sum }}\;\overset{M=8}{\underset{\forall X}{\sum }}\frac{{SAR}_{\text{healthy tissue}}\left(f,X\right)}{{SAR}_{\text{tumor}}\left(f,X\right)}, \end{array}$$

where SAR_tumor_ and SAR_healthy  tissue_ indicate the spatial integration of absorbed SAR in tumor and healthy tissue, respectively. *f* denotes the desired frequency bandwidth ([−*B*/2, *B*/2]) for which EM hyperthermia system is evaluated. *M* is the number of antenna applicators deployed.

Optimization is simplified by assuming discrete values for magnitude and phase. The magnitude Λ is normalized from 0 to 1with a step of 0.1, and the phase Θ is chosen with a step of three degrees. The optimization problem becomes

(7) Minimize: ξM,f,Xwhere  M=8,  f∈−B2,B2,  X∈Λ,  Θsubject  to: 0≤Λ<1, 0°≤Θ<360°.

**Figure 4:**
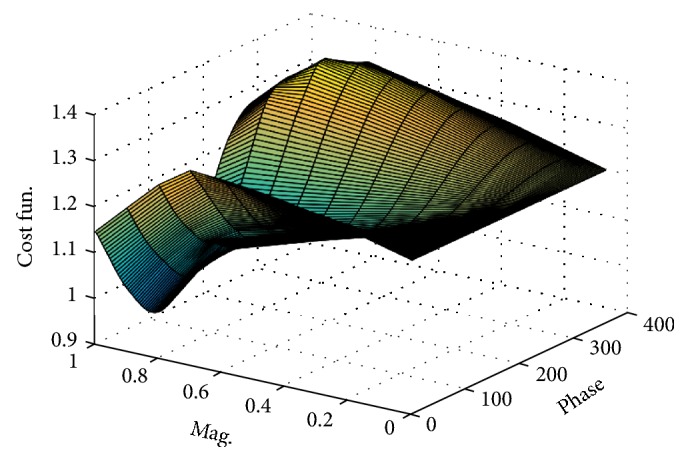
Variation of cost function in terms of phase and magnitude.

Figure 4 shows the global optimum phase and magnitude values of the cost function *ξ*(*M*, *f*, *X*), plotted as a function of magnitude and phase for the first port.

As noticed from Figure 4, global optimum magnitude and phase values for which cost function is minimized are achieved for **X** ∈ (0.8, 306°).

### 3.2. Time Reversal (TR) Based Energy Focusing

With the advantage of being fast, time reversal (TR) compared to SAR or temperature optimization techniques allows the use of sinusoidal or pulse form of the excitation signal to focus energy at tumor site. TR allows the estimation of phase and magnitude of E-fields from wave simulation and not from measured data [50]. Typically, TR runs in two steps [56, 57]. In the first step, a virtual energy source is placed at the desired focusing location and the wavefront of the source is propagated through the inhomogeneous head model. The propagated fields are recorded at phantom surface. The fields are acquired and processed in a time-reversed order. In frequency domain, this corresponds to the conjugate of recorded signals. In step two, the virtual source is removed and antenna applicators or transducers are placed at points of maximum field and are driven by the time-reversed signals. It is because of the time invariance characteristics of EM or US field that the back-propagated time-reversed fields are capable of refocusing at the initial position of virtual source. Here in this research, we use a plane of virtual source at the tumor center. This plane has a dimension of 1 cm × 1 cm and can be considered as a grid of point sources. Since our model in EM hyperthermia case is 3D, therefore, we choose three such planes covering the tumor in *xy*, *yz*, and *xz* directions.

Because of linearity, the field measured by an applicator/transducer at the phantom surface from the* m*th excitation point is given by

(8) ft∑m=1Mhmt⊗emt=∫−∞+∞∑m=1Mhmtemt−τdτ,

where ⊗ represents the temporal convolution and *h*_*m*_(*t*) is the linear propagation operator defined by Green's function. This term includes the propagation effects of the medium from the *m*th excitation point to the transducer/applicator. *e*_*m*_(*t*) corresponds to the excitation signal of point source and *f*(*t*) is the signal measured by the transducer. In frequency domain, the above equation can be written in matrix form as follows:

(9) $$\begin{array}{c} F\left(\;\omega \;\right)=H_{m}\left(\;\omega \;\right)E_{m}\left(\;\omega \;\right). \end{array}$$

Here, the matrix *H*_*m*_(*ω*) is the Fourier transform of Green's function. By assuming spatial reciprocity condition, the propagation between the transducer and virtual point sources can be represented as

(10) $$\begin{array}{c} E_{m}\left(\;\omega \;\right)={H_{m}}^{T}\left(\;\omega \;\right)F\left(\;\omega \;\right). \end{array}$$

Here, *H*_*m*_^*T*^ is the transpose of *H*_*m*_. For the time reversal step, the time reversal operation of Green's function [*h*_*m*_(−*t*)] in time domain is equivalent to complex conjugate in frequency domain. Therefore, the time reversing the spectrum of the transducer's output yields

(11) $$\begin{array}{c} F^{*}\left(\;\omega \;\right)={H_{m}}^{*}\left(\;\omega \;\right){E_{m}}^{*}\left(\;\omega \;\right)={H_{m}}^{*}\left(\;\omega \;\right)E_{m}\left(\;\omega \;\right). \end{array}$$

Here, *E*_*m*_ is real and “*∗*” represents the complex conjugate. By combing the previous two equations, the back-propagated signal at the initial source location can be achieved as follows:

(12) ETRωHmTωF∗ω=HmTωHm∗ωEmω,

where *H*_*m*_^*T*^(*ω*)*H*_*m*_^*∗*^(*ω*) is known as the time reversal (TR) operator.

Here, a time reversal (TR) tool is developed using MATLAB [58], which has the capability to interface with CST and COMSOL simulating environments to automate the process of field acquisition, processing, and feeding back to the applicators and transducers in simulation environments.

## 4. Simulation Results

The next subsections present the simulation results of the adopted energy localization techniques of the proposed hybrid system. They include the energy localization results for EM and US hyperthermia modules along with the synergetic effect of both treatment modalities in terms of temperature optimization.

### 4.1. EM Hyperthermia Results

**Figure 5:**
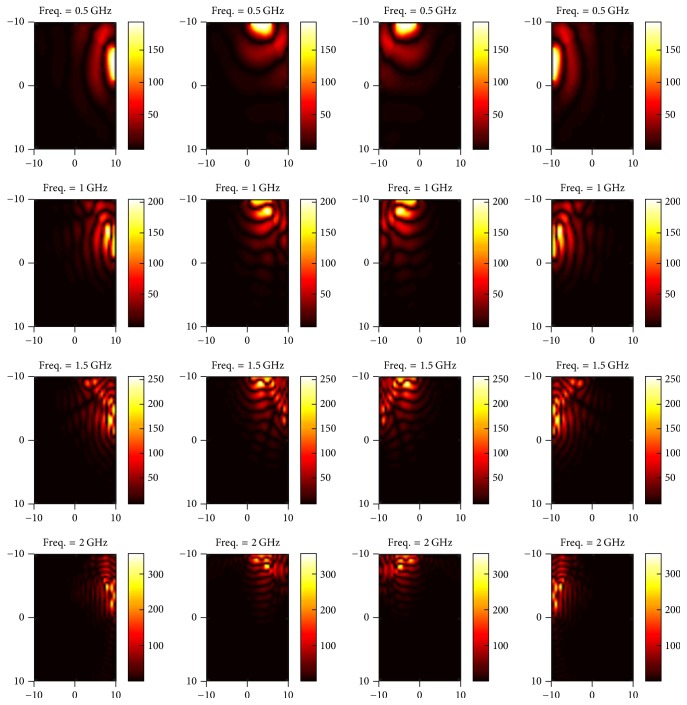
E-field map of excitation ports numbered 1–4 (left to right) for different subcarriers (top to bottom).

**Figure 6:**
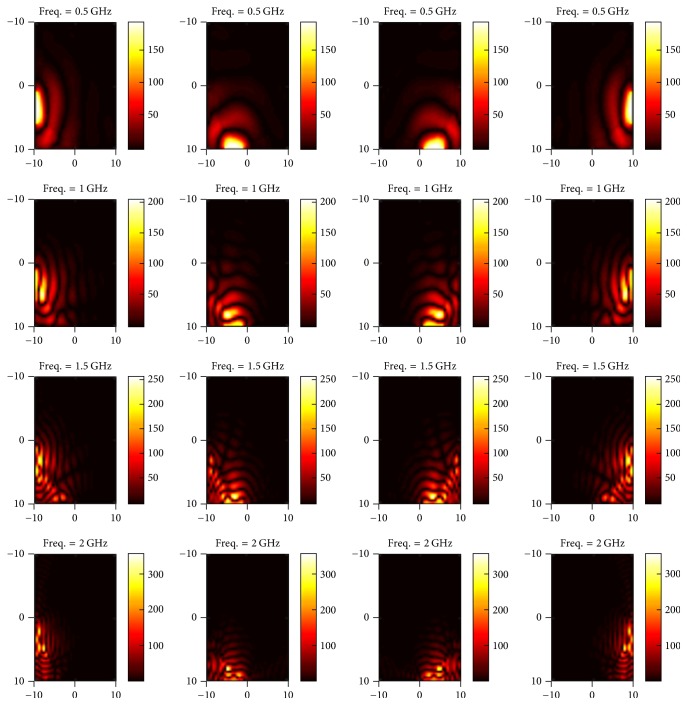
E-field map of excitation ports numbered 5–8 (left to right) for different subcarriers (top to bottom).

In this section, we present simulated results for our proposed multichannel wideband EM hyperthermia system. The first phase of the results is associated with the instance when applicator ports are excited with different subcarriers such as 0.5, 1, 1.5, and 2 GHz. The E-field maps in *z* direction (towards tumor center) are shown in Figures 5 and 6.

**Figure 7:**
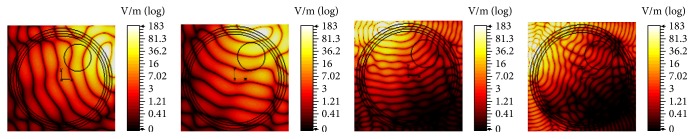
E-field map of EM energy propagating towards the tumor when ports 1–4 from left to right are excited by frequency subcarriers of 1, 1.5, 2, and 2.5 GHz, respectively.

**Figure 8:**
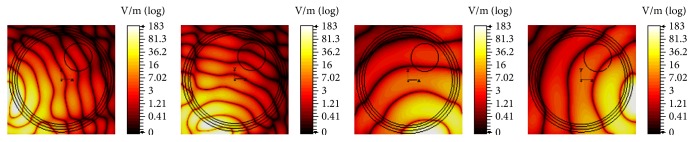
E-field map of EM energy propagating towards the tumor when ports 5–8 from left to right are excited by frequency subcarriers of 3, 2.75, 0.75, and 0.5 GHz, respectively.

In the second phase, we demonstrate EM energy propagation towards the tumor region from each port under different excitation frequencies as shown in Figures 7 and 8.

**Figure 9:**
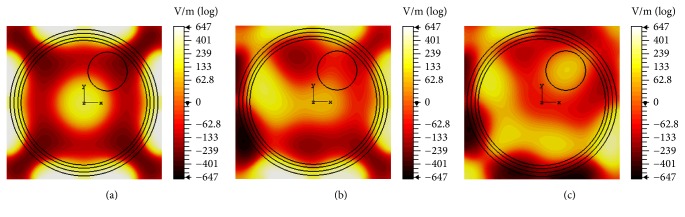
The capability of coherent-phased-array tool to localize energy for (a) zero phase shift, (b) inappropriate phase shift, and (c) appropriate phase shift when frequency of operation is 0.5 GHz.

From Figures 7 and 8, it is evident that more energy is accumulated in the ports' vicinity than in the tumor region; therefore, we devise a coherent-phased-array tool to localize energy at the tumor site [59]. By choosing appropriate dielectric properties of human head tissues for 0.5 GHz derived from [48, 49], the performance of the phased-array tool is investigated for various phase shifts. The purpose of this phased-array tool is to achieve constructive interference of EM energy at the tumor region and destructive interference in the surrounding tissue. To achieve this goal, appropriate phase shifts are chosen by taking into consideration factors such as phase, amplitude, the number of subcarriers, and the propagated distances of subcarriers from the ports to the target region. The results achieved after incorporation of the phased-array tool are shown in Figure 9.

With no phase shift, energy concentration is achieved at the phantom center shown in Figure 9(a). This can prove an ideal energy localization, had the tumor been placed at the center. With some arbitrary phase shift, the focus of EM energy shifts to some off-target position presented in Figure 9(b). With a correct phase shift, good energy focus is achieved at tumor location depicted in Figure 9(c). However, we notice that focusing of energy onto the intended region is associated with generation of hotspots because of the unnecessary heating of surrounding healthy tissue. This is shown on the left of tumor in Figure 9(c).

**Figure 10:**
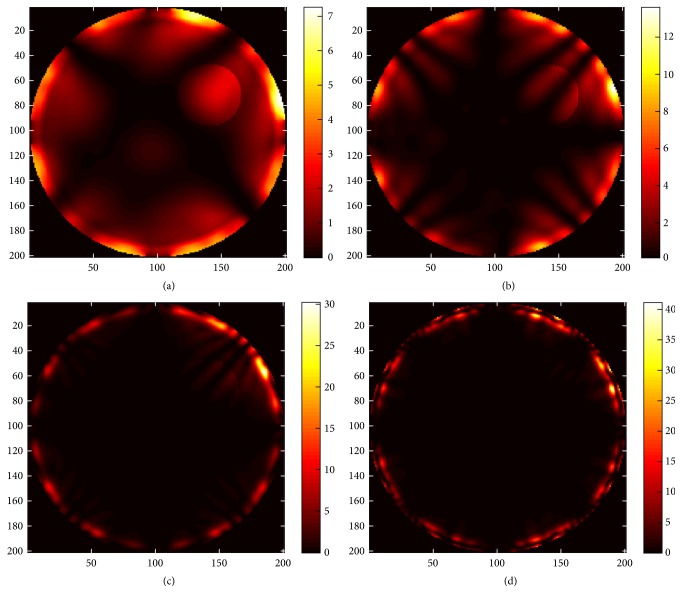
SAR optimization for the narrowband case when frequency is (a) 0.5 GHz, (b) 1 GHz, (c) 2 GHz, and (d) 3 GHz.

In order to reduce the hotspots that resulted from the phased-array tool, we propose SAR optimization based on the wideband genetic algorithm technique. The performance of the optimization tool is investigated for different frequency bandwidths of operation. Figures 10(a), 10(b), 10(c), and 10(d) show SAR optimization for the narrowband (single frequency) case when frequency is 0.5, 1, 2, and 3 GHz, respectively.

**Figure 11:**
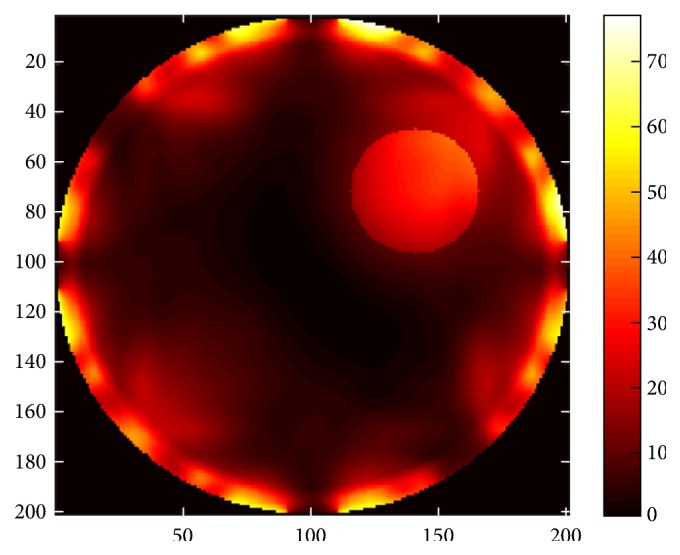
SAR optimization for the wideband (0.3–3 GHz) case.

As illustrated in Figure 10, the penetration depth of EM energy decreases as frequency increases. Therefore, we increase frequency bandwidth (0.3–3 GHz) and invoke SAR optimization to notice an improvement in energy localization. The overlaying effect of SAR for a band of frequencies (0.3−3 GHz) is illustrated in Figure 11.

**Figure 12:**
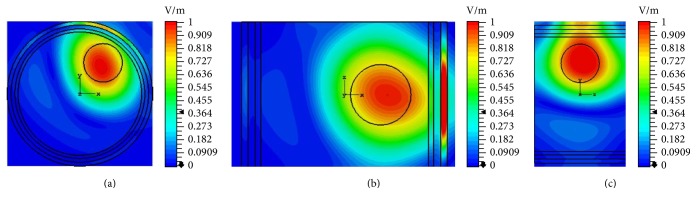
TR focusing for EM hyperthermia in (a) *xy*, (b) *xz*, and (c) *yz* planes when frequency of operation is 0.5 GHz.

It can be concluded that, with increased bandwidth and multichannel techniques, deposition of EM energy can be maximized at tumor locations. Furthermore, to investigate the performance of EM hyperthermia in TR domain, we choose a spatial plane of excitation source at the tumor center. The fields are captured at the surface of the phantom in different planes, time-reversed, and propagated back to the tumor center. The results of TR focusing are shown in Figure 12.

### 4.2. US Hyperthermia Results

**Figure 13:**
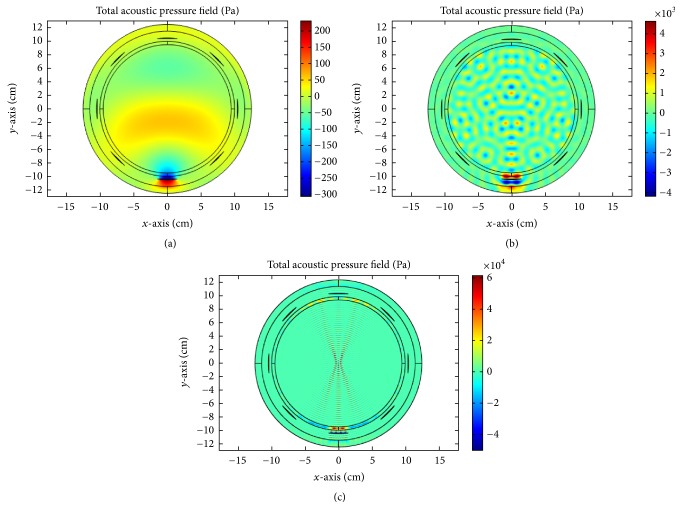
Single transducer insonation for different frequencies: (a) 0.05 MHz, (b) 0.1 MHz, and (c) 0.5 MHz.

In this section, we discuss the acoustic and thermal simulation results of US hyperthermia module. The use and design of the acoustic transducer are deduced from [60]. We use two independent pressure sources within one single transducer and phases are adjusted based on the coherent-phased-array concept. First, we investigate the propagation of ultrasound energy inside the human head by exciting one of the transducer elements with frequencies of 0.05, 0.1, and 0.5 MHz, respectively. This is shown in Figures 13(a), 13(b), and 13(c). Comparatively sharp focusing is achieved with reference frequency (0.5 MHz) by using a single transducer.

**Figure 14:**
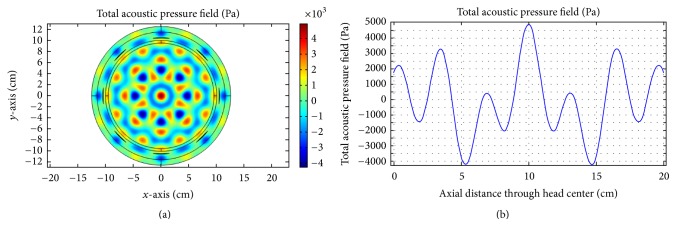
(a) Acoustic field intensity in the absence of skull-bone layer and (b) the corresponding pressure at axial distance through the head center at 0.05 MHz.

**Figure 15:**
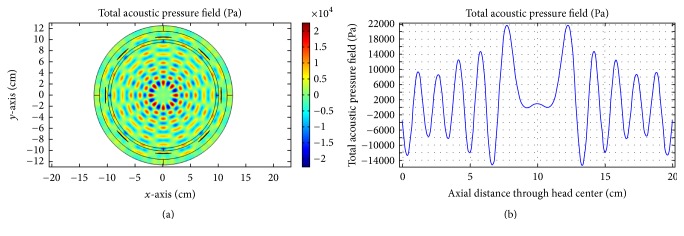
(a) Acoustic field intensity in the absence of skull-bone layer and (b) the corresponding pressure at axial distance through the head center at 0.1 MHz.

**Figure 16:**
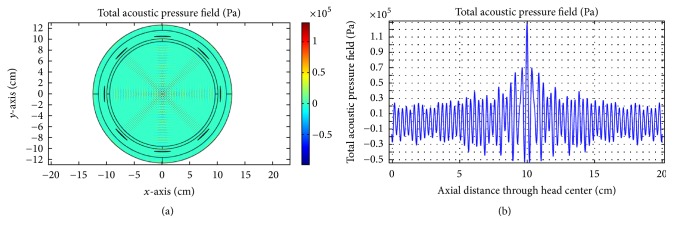
(a)Acoustic field intensity in the absence of skull-bone layer and (b) the corresponding pressure at axial distance through the head center at 0.5 MHz.

For multitransducer excitation, we consider two cases. For the first case, the insonation process is evaluated when there is no skull-bone layer. Because of phantom symmetry, the ultrasound energy should focus at the head center. In order to measure the total transducer pressure inside the brain tissue, an axial reference line is drawn through the center of the phantom. The insonation results and the corresponding acoustic pressure at the brain center for no skull-bone layer are presented in Figures 14–16.

**Figure 17:**
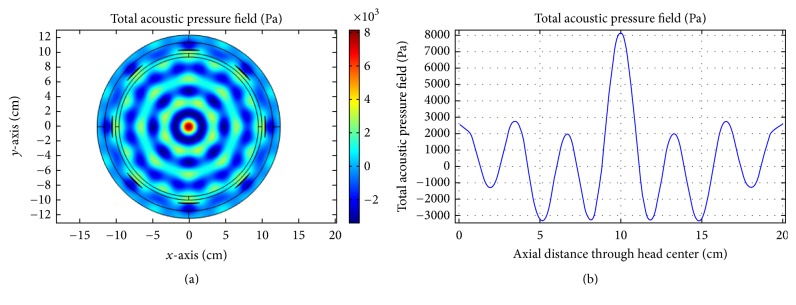
(a) Acoustic field intensity in the presence of skull-bone layer and (b) the corresponding pressure at axial distance through the head center at 0.05 MHz.

**Figure 18:**
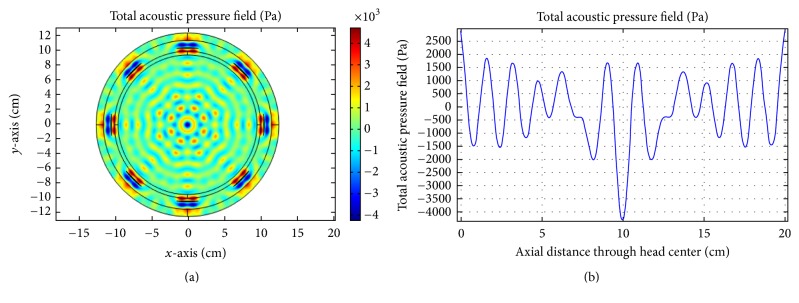
(a) Acoustic field intensity in the presence of skull-bone layer and (b) the corresponding pressure at axial distance through the head center at 0.1 MHz.

**Figure 19:**
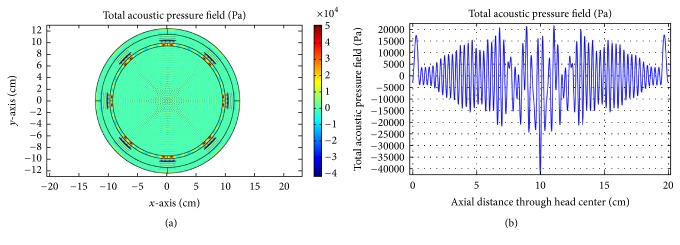
(a) Acoustic field intensity in the presence of skull-bone layer and (b) the corresponding pressure at axial distance through the head center at 0.5 MHz.

As obvious from Figure 16, good focusing and corresponding high acoustic pressure are achieved for 0.5 MHz at the head center. For the second case, we incorporate the skull-bone layer and investigate the effect of insonation for all transducers. The results are shown in Figures 17–19.

**Figure 20:**
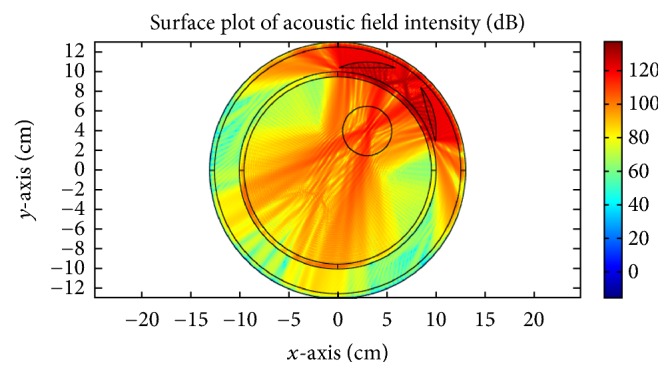
Localization of acoustic energy at the tumor center using two transducers with a mechanical approach.

From the figures, it is apparent that more acoustic energy is absorbed in the skull-bone layer and minimum energy approaches the head center and the corresponding acoustic pressure at the head center varies accordingly. The results achieved should be ideal, had the tumor been located at the center. Since in our case the tumor is positioned at the top right, therefore to achieve energy localization we adopt two approaches classified as mechanical and electronic. The electronic approach is based on TR focusing by taking the excitation of 256-element transducers into account. In the mechanical approach, we aim to use the minimum number of transducer elements, two in this case, to achieve reasonable focus at the tumor center. The optimum locations for placing these two transducers are deduced from the TR approach. The acoustic response of the mechanical approach is shown in Figure 20.

As apparent from Figure 20, the acoustic focus matches the tumor center by mechanically deploying two transducers. In order to implement the TR approach, we place a point source at the tumor center. For a source to be uniform and to radiate equally strong in all directions, we choose a monopole point source given by

(13) $$\begin{array}{c} P=\frac{4\pi }{\rho_{c}}S\delta \left(\;x-x_{0}\;\right)\left(\;y-y_{0}\;\right), \end{array}$$

where *δ*(*x* − *x*_0_)(*y* − *y*_0_) represents the delta function in 2D space and adds a source at a point (*x*, *y*) = (*x*_0_, *y*_0_). Here, this point corresponds to (3, 4) and *ρ*_*c*_ is the mass density. Here, we characterize *S* as volume flow rate per unit length out from source *Q*_*s*_ and it is given by

(14) $$\begin{array}{c} S=e^{i\emptyset }\frac{i\omega \rho_{c}Q_{s}}{4\pi }. \end{array}$$

**Figure 21:**
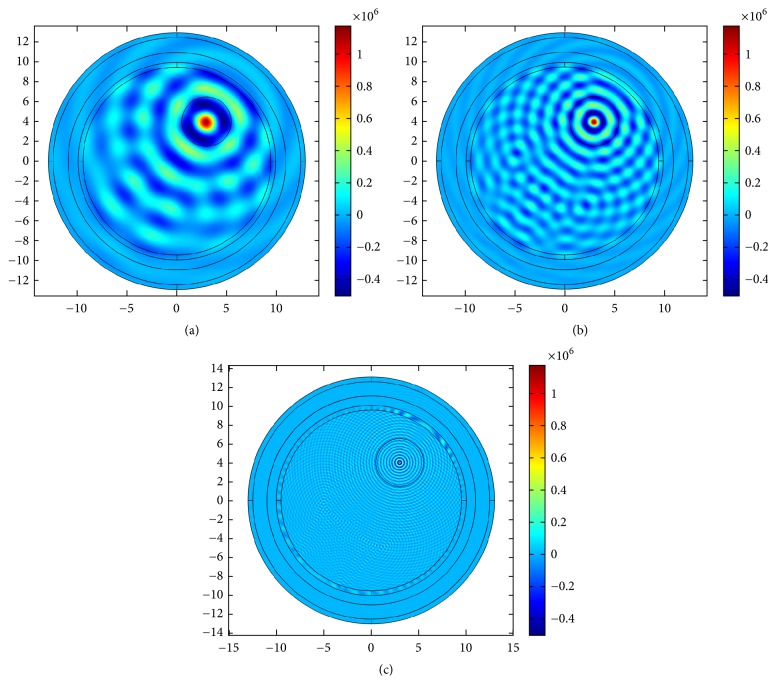
Step 1 of TR localization for US hyperthermia when a monopole source is excited with frequencies of (a) 0.05 MHz, (b) 0.1 MHz, and (c) 0.5 MHz each normalized to 1 MPa.

**Figure 22:**
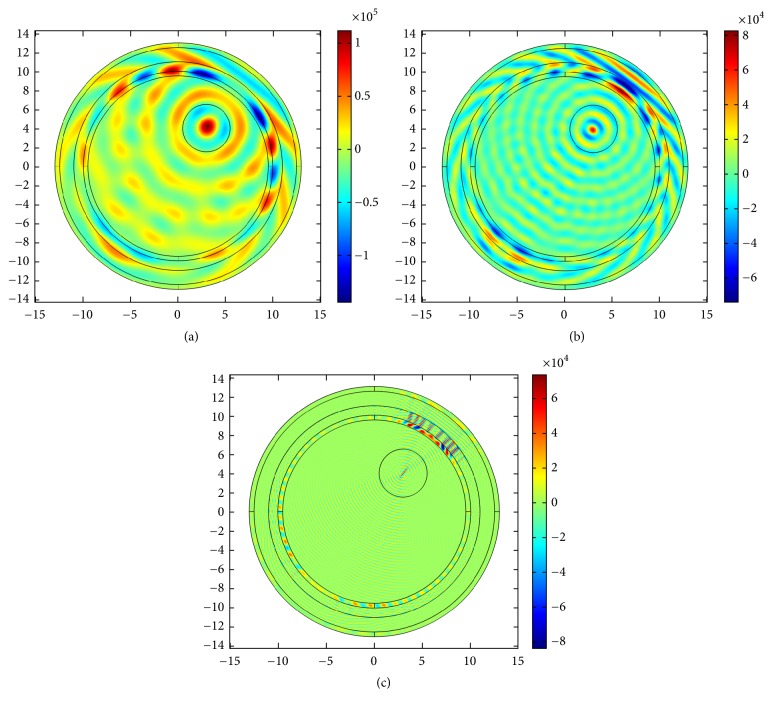
Step 2 of TR localization for US hyperthermia when acoustic energy from transducers is back-propagated towards the tumor with frequencies of (a) 0.05 MHz, (b) 0.1 MHz, and (c) 0.5 MHz.

A circular transducer array of 256 elements surrounds the head. These transducers record the fields from monopole source excitation. The developed TR tool processes recorded fields and feeds them back to the transducers. The output pressure of these transducers is normalized to 1 MPa and the results are shown in Figures 21 and 22, where Figure 21 shows the results for step 1, when the monopole source is excited with frequencies of 0.05, 0.1, and 0.5 MHz, respectively, and Figure 22 depicts step 2 of the TR as the fields are back-propagated to refocus at the tumor center.

To investigate the thermal response of the two approaches, we follow the two-dimensional bioheat transfer equation given by

(15) $$\begin{array}{c} \frac{\partial^{2}T}{\partial x^{2}}+\frac{\partial^{2}T}{\partial y^{2}}+\frac{W_{b}C_{b}}{K}\left(\;T_{b}-T\;\right)=\frac{Q\left(x,y\right)}{K}. \end{array}$$

**Figure 23:**
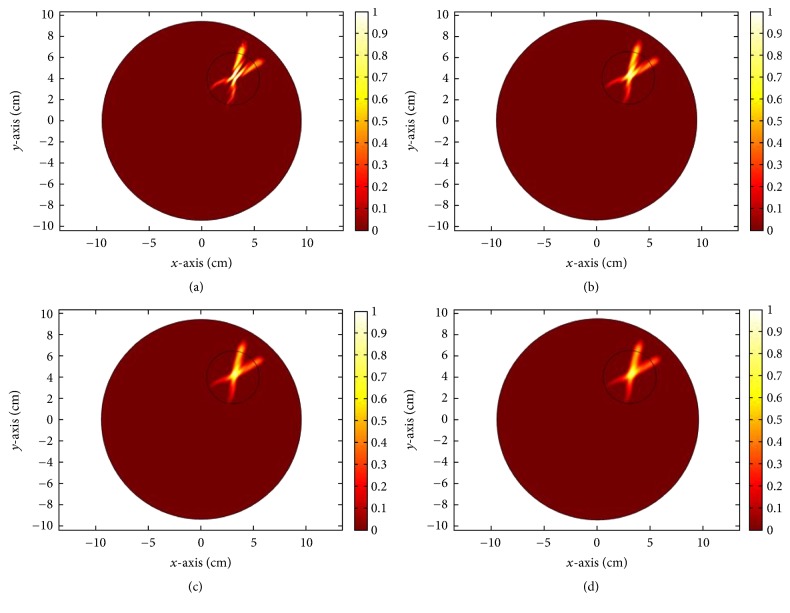
Variation in thermal intensity (normalized) at the tumor center for different time insonations, (a) 2 sec, (b) 6 sec, (c) 10 sec, and (d) 14 sec, for mechanical alignment focusing approach.

**Figure 24:**
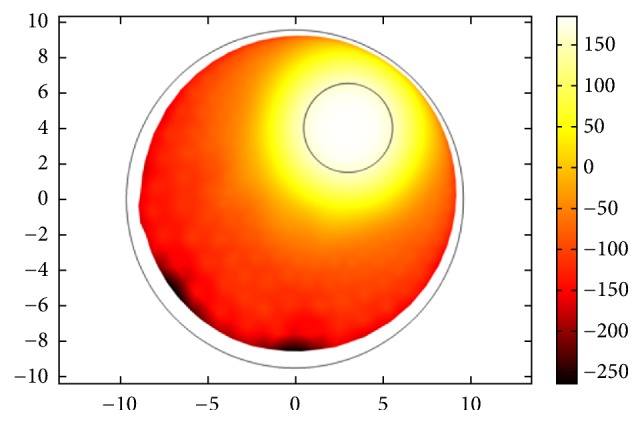
Thermal response (log scale) of the proposed US hyperthermia module based on TR focusing approach.

*K* is the thermal conductivity, *T* is the temperature at any point (*x*, *y*), *W*_*b*_ = 8 Kg/m^3^/s describes blood perfusion, *C*_*b*_ = (4000 J/Kg)/C° denotes specific heat of blood, and *T*_*b*_ = 37°C represents blood temperature. *Q* is the heat absorption determined by the acoustic field intensity *I*(*x*, *y*). The results in Figure 23 correspond to mechanical alignment focusing approach. It is obvious that as the time for insonation increases, more of the tumor concentric region is heated.  Figure 24 illustrates the thermal response using log scale of US hyperthermia module when adopting the TR focusing approach.

### 4.3. Synergetic Effect of EM and US Hyperthermia

Combination of therapeutic performances of different treatment modalities is challenging because of the differences in physical and biological mechanisms associated with treatment modalities. However, recent research shows a new trend in combining healing potentials of different treatment techniques. This includes the effective treatment of focused ultrasound (FUS) in combination with gamma knife discussed in [61]. The simultaneous action of radiation and heating on enhancing the therapeutic effect is analyzed in [62]. Simultaneous delivery of intraoperative radiation and ultrasound hyperthermia is studied in [63]. Progress in hardware tools also supports the augmentation of various energy based subsystems. Excitation can depend on digital wave shaping of signals using arbitrary waveform sources. Acquisition can also depend on high-speed analog-to-digital converters. The main control is obtained using microprocessor and FPGA based systems.

**Figure 25:**
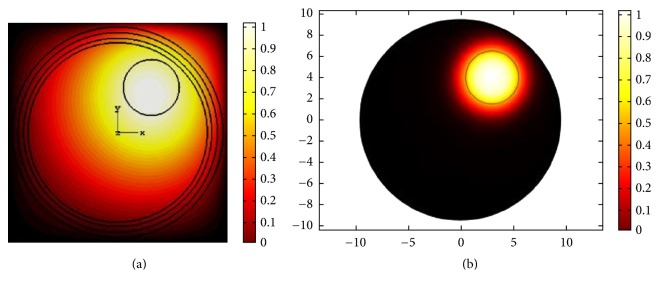
Normalized temperature profiles of (a) EM hyperthermia module and (b) ultrasound hyperthermia module based on time reversal (TR) localization.

For the proposed hybrid EM and US hyperthermia treatment system, we choose TR based temperature optimization. The normalized thermal maps of both modalities for treating cancerous head tumors are shown in Figure 25. The synergetic effect would be superposition of thermal profiles of both modalities. From the figure, it is obvious that US hyperthermia gives highly focused response targeting small area as compared to EM. EM hyperthermia on the other hand is beneficial when heating a large volumetric target. The simultaneous action of both therapies can lead to enhanced treatment of deep-seated tumors.

## 5. Discussion and Conclusions

EM and US fields are typically used in noninvasive thermal treatment systems. EM energy excitation can be planned using wideband subsystems that include a waveform shaping module, a power amplifier, and an antenna applicator. The resulting excitation field pattern is wide, allowing the exposure of large areas to the applied energy. The size of the antenna applicator elements allows the operation with few elements in phased-array configuration to control steering EM energy to tumor locations. On the other hand, US transducers are small, allowing the development of an array with a large number of elements. The much smaller wave propagation speed of US signal in tissue compared to EM fields provides shorter wavelength and allows targeting small sized tumors. Different transducer element sets can be chosen with the capability to operate at different frequency values to provide reasonable degrees of freedom in controlling energy excitation. In addition, US energy has better penetration into human tissues and can thus reach deep-seated tumors.

The approach adopted in this paper is to develop models that can be easily adjusted to patient data to be obtained from MRI or CT scan images. With appropriate dielectric and acoustic properties of different tissues, qualitative analysis of a near-real-time thermal treatment is achieved. Different techniques of energy localization for both modalities are discussed. A genetic algorithm optimization tool is developed to optimize the waveform shaping of each of the excitation ports resulting in maximizing SAR at tumor while eradicating hotspots. The proposed wideband SAR optimization technique gives more degrees of freedom in choosing different frequency bandwidths, which alternatively gives more advantages over conventional single frequency optimization in terms of energy penetration depth and localization of energy. The TR focusing approach is implemented in EM and US modules to study the synergetic effect. The presented analysis and the associated results suggest that the proposed hybrid system can present a new paradigm in hyperthermia treatment of brain tumors.

## Figures and Tables

**Table 1:** Acoustic properties of various head tissues at 0.5 MHz [48, 49].

Material	Density (*ρ*)	Speed (*c*)	Attenuation (*α*)
	Kg/m^3^	m/s	N_p_/m
Skin	1050	1500	6.5
Brain	1043	1510	5.5
Skull bone	1900	4080	76
Water	1000	1480	0.025
Tumor	1050	1500	5
